# Geographical and temporal trends and seasonal relapse in *Plasmodium ovale* spp. and *Plasmodium malariae* infections imported to the UK between 1987 and 2015

**DOI:** 10.1186/s12916-018-1204-6

**Published:** 2018-11-27

**Authors:** Laura E. B. Nabarro, Debbie Nolder, Claire Broderick, Behzad Nadjm, Valerie Smith, Marie Blaze, Anna M. Checkley, Peter L. Chiodini, Colin J. Sutherland, Christopher J. M. Whitty

**Affiliations:** 1 Public Health England Malaria Reference Laboratory, London School of Hygiene and Tropical Medicine, Keppel Street, London, WC1E 7HT UK; 2 The Hospital for Tropical Diseases, Mortimer Market Capper Street, London, WC1E 6JD UK; 3 Oxford University Clinical Research Unit, Hanoi, Vietnam

**Keywords:** Malaria, Ovale, Malariae, Season, Imported, Relapse, Temporal, Geographical, Vivax, Traveller

## Abstract

**Background:**

*Plasmodium ovale* spp. and *P. malariae* cause illness in endemic regions and returning travellers. Far less is known about these species than *P. falciparum* and *P. vivax.*

**Methods:**

The UK national surveillance data, collected 1987 to 2015, were collated with the International Passenger Survey and climatic data to determine geographical, temporal and seasonal trends of imported *P. ovale* spp*.* and *P. malariae* infection.

**Results:**

Of 52,242 notified cases of malaria, 6.04% (3157) were caused by *P. ovale* spp. and 1.61% (841) by *P. malariae*; mortality was 0.03% (1) and 0.12% (1), respectively. Almost all travellers acquired infection in West or East Africa. Infection rate per travel episode fell fivefold during the study period. The median latency of *P. malariae* and *P. ovale* spp. was 18 and 76 days, respectively; delayed presentation occurred with both species. The latency of *P. ovale* spp. infection imported from West Africa was significantly shorter in those arriving in the UK during the West African peak malarial season compared to those arriving outside it (44 days vs 94 days, *p* < 0.0001), implying that relapse synchronises with the period of high malarial transmission. This trend was not seen in *P. ovale* spp. imported from East Africa nor in *P. malariae.*

**Conclusion:**

In West Africa, where malaria transmission is highly seasonal, *P. ovale* spp. may have evolved to relapse during the malarial high transmission season. This has public health implications. Deaths are very rare, supporting current guidelines emphasising outpatient treatment. However, late presentations do occur.

## Background

*Plasmodium ovale* spp*.* and *P. malariae* malaria are less common than *P. falciparum* and *P. vivax* and cause a milder clinical syndrome than *P. falciparum*. As a result, much less is known about them. Although mostly found in Africa, they are both widely distributed through tropical areas of Asia and Australasia and *P. malariae* is also found in the Americas. They are important causes of febrile illness in these regions and in returning travellers. Their prevalence is often underestimated due to characteristically low parasitaemias and difficulty in distinction from other malaria species by light microscopy [1]. Recent molecular studies have suggested that these infections are more common than previously thought and are often mixed with *P. falciparum* [2–4].

In Africa, *P. ovale* spp*.* are the principal relapsing forms of malaria. *P. malariae* may also cause long-lasting asymptomatic infection using mechanisms that are not yet certain [5]. The ability of these species to remain undiagnosed for long periods before multiplication and gametocytaemia occur may hinder malaria eradication efforts in endemic areas. It also suggests that they are likely to increase as a proportion of malaria as control efforts improve.

Both species are also important causes of febrile illness in returning travellers because, unlike *P. falciparum*, they may present months or even years after return from an endemic region [5, 6]. At this point, patients and their physicians are less likely to link a febrile illness with travel, resulting in delayed or missed diagnosis [7].

In recent years, *P. ovale* has been distinguished into two separate species, *P. ovale curtisi* and *P. ovale wallikeri*. These two species, though morphologically identical by light microscopy, are genetically distinct and non-recombinant. Both species are thought to form hypnozoites and to be sympatric throughout their geographical range [8]. It is unclear whether clinical disease differs between the two species [9, 10].

This study, which is the largest series of *P. ovale* spp. and *P. malariae* infections presented to date, aims to answer three sets of questions. Firstly, how do these species present in non-endemic countries and what are the travel and temporal trends? Secondly, is there any difference in seasonal latency in *P. ovale* spp. infections, as is seen in *P. vivax* malaria, the other relapsing malarial species? Thirdly, using a subset of isolates speciated to either *P. ovale curtisi* or *P. ovale wallikeri,* is there any difference in latency between the two?

## Methods

The Public Health England Malarial Reference Laboratory (PHE MRL), formerly the Health Protection Agency Malarial Reference Laboratory, has been systematically collecting malaria data in the UK since 1987. This represents one of the largest and most complete continuous series on imported malaria worldwide. The process has been previously described, and in order to maintain comparable data over time, methodology has remained largely unchanged since its inception [11]. Inclusion is dependent on parasitological diagnosis by blood film or molecular methods undertaken in a specialist malaria laboratory. Passive surveillance is conducted by statutory notification of all cases from UK hospitals. UK laboratories also send blood films for verification of diagnosis by light microscopy and polymerase chain reaction. Data from reporting hospitals are collected on a standardised form; this includes age, sex, countries visited, reason for and dates of travel. A capture-recapture study estimates a national case ascertainment rate of 56% [11].

All cases of *P. ovale* spp*.* and *P. malariae* reported between January 1, 1987, and December 31, 2015, were identified. Once cleaned, the data were analysed for geographic, seasonal and temporal trends. Countries were grouped as per United Nations’ regions. This large dataset includes some cases considered in two previous studies examining latency and prophylaxis use [5, 6].

The study set out to identify any seasonal trends in latency, in particular whether there was evidence of a tendency for *P. ovale* spp. to relapse during the period of peak malaria transmission in region of acquisition. The period of high malaria transmission in West Africa (the malaria season) was defined as August to November using data from studies conducted to inform seasonal malarial chemoprevention [12–15]. Although malaria is less seasonal in East Africa, the malaria season was designated as April to July inclusive. The latent period was defined as time between arrival in the UK and onset of symptoms.

Data on travel were collated with the International Passenger Survey (IPS). This continuous survey uses 250,000 interviews per year from travellers at UK sea, land and air ports to estimate travel to and from the UK to individual countries [16]. Pertinent data were available from 1993 onwards. These data were used as a denominator to establish attack rates and change in incidence of infection in travellers over time. In contrast to the methodology used for primary collection of the malaria data, which aims to be complete, the IPS is a representative sampling methodology. The two databases were not combined.

Prior to 2003, light microscopy alone was used for species identification. Conventional PCR to detect species was introduced in 2003. Since 2013, some *P. ovale* spp. samples have been further speciated to *P. ovale curtisi* and *P. ovale wallikeri* using molecular methods. As previously described [6], *P. ovale* spp. infections are first confirmed by conventional amplification of the small subunit ribosomal RNA genes [17, 18]. The two species are then discriminated by conventional amplification at the potra locus and quantitative real-time PCR amplification of the porbp2 locus [19]. These data were further analysed to compare geographic, seasonal and temporal trends between these two species.

Data were entered using dBase and analysed using STATA 14. Simple proportions were calculated together with rates of infection. Wilcoxon rank-sum was used to establish significance for skewed continuous data, mainly latency.

This study analyses routine surveillance data collected, analysed and published as the legal obligation of PHE under Regulation 3 of the Health Service Regulations 2002. Such analysis does not have separate ethics approval but is covered under existing regulatory frameworks and responsibilities. No extra data requiring separate ethics approval were collected as part of this study. Data from individual patients were pseudo-anonymized before analysis.

## Results

**Fig. 1:**
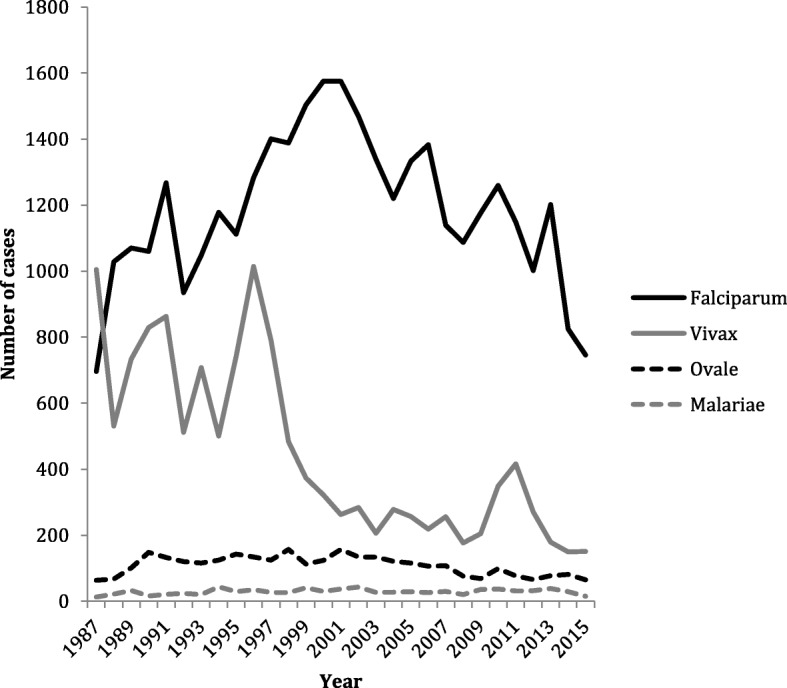
Cases of malaria by species notified each year, 1987–2015

Between January 1, 1987, and December 31, 2015, 52,242 cases of malaria were notified to the MRL: 59.7% (31,191) cases were male and 35% (18,273) female. Sex was not documented in 5.3% (2778) cases. The median age was 32 years (interquartile (IQ) range 22–45). *P. falciparum* was the most common imported species (34,455, 66.0% of notifications), followed by *P. vivax* (13,070, 25.0%), *P. ovale* spp. (3157, 6.0%), *P. malariae* (841, 1.6%) and *P. knowlesi* (1, 0.002%). There were 588 (1.1%) mixed species infection and 162 cases (0.3%) where the species was not documented. Figure 1 shows the speciated cases of malaria notified each year since 1987. When proportions of cases before the year 2000 were compared with those after 2000, the proportion of *P. vivax* had fallen by 44.5% whilst the proportion of *P. falciparum*, *P. malariae* and *P. ovale* spp. had risen by 32%, 41% and 5.7%, respectively.

### Demographic and clinical features

#### *P. ovale* spp.

Three thousand one hundred fifty-seven cases of *P. ovale* spp. were notified over this period: 65.2% (2058) were male and 30% (947) female (4.8%, 152 sex not documented) with a median age of 28 (IQ range 21–38). In 86.7% (2738) cases, the region of acquisition was documented. Of these, 98.2% (2689) cases of *P. ovale* spp. were acquired in Africa, mostly from West (58.4%, 1599) and East Africa (24.6%, 675) but also from Central (5.5%, 150), North (0.7%, 20) and South (1.2%, 32) Africa. Two hundred thirteen people (7.8%) had travelled to more than one region in Africa, so the region of malaria acquisition was unclear. The commonest countries where infection was acquired were Nigeria (33.3%, 911), Ghana (12.2%, 335), Uganda (7.3%, 199), Kenya (6.1%, 167), and Sierra Leone (5.6%, 154). Most people acquiring infection in West Africa were visiting friends and family (87.3%, 577) whilst those acquiring infection in East Africa were predominantly tourists (55.5%, 142, *p* < 0.001). The mortality rate was 0.03%(1); the only recorded death occurred in a 51-year-old woman who died of splenic rupture, a direct complication of malaria but one which occasionally occurs with all species. In contrast to *P. vivax*, where deaths are heavily weighted to older patients [20], no deaths were reported in the 135 (4.4%) patients aged more than 60 years.

The median latency from arrival was 76 days for *P. ovale* spp. (range − 53 to 3167 days), compared to 4 days for *P. falciparum* and 67 days for *P. vivax*. Delayed presentation, meaning presentation beyond 90 days, occurred in 23.4% (738) cases of *P. ovale* spp*.* with 2.7% (85) cases presenting more than a year after arrival in the UK. In comparison, 0.6% (197) of *P. falciparum* cases presented more than 90 days after arrival in the UK and 0.1% (42) after more than a year: 22.0% (2879) of *P. vivax* cases presented more than 90 days after arrival and 2.1% (269) after more than a year.

#### *P. malariae*

Eight hundred forty-one cases of *P. malariae* were notified over this period; 63.7% (536) were male, and 31.4% (264) were female (4.9%, 41 sex not documented) with a median age of 29 (IQ range 20–40). In 86.0% (723) cases, the region of acquisition was documented. 98.5% (712) infections were acquired in Africa, mostly from West (55.9%, 404) and East Africa (28.9%, 209) but also from Central (6.5%, 47), North (1.1%, 8) and South Africa (0.4%, 3). Forty-one people (5.7%) had travelled to more than one region in Africa, so the region of malaria acquisition was unclear. The commonest countries of acquisition were similar to those of *P. ovale* spp.: Nigeria (30.7%, 222), Ghana (12.6%, 91), Uganda (10.9%, 79), Kenya (8.4%, 61) and Sierra Leone (4.7%, 34). Most people acquiring infection in West Africa were visiting friends and family (91.2%, 175) whilst those acquiring infection in East Africa were evenly split between those visiting friends and family (48.8%, 41) and tourists (51.2%, 43, *p*< 0.001). The mortality rate was 0.12% (1). The single death was that of a 48-year-old man who died of a hospital-acquired pneumonia, a complication of hospital admission rather than a recognised direct complication of *P. malariae* malarial infection. There were no deaths reported in 29 patients (3.5%) aged more than 60 years.

As previously reported [5], the median latency of *P. malariae* was 18 days (IQ range 3–46, range − 51 to 1123 days). 4.2% (35) cases presented more than 90 days after arrival in the UK, and 0.2% (2) cases presented more than a year after arrival.

### Rates of infection compared to travel data—temporal trends

**Fig. 2:**
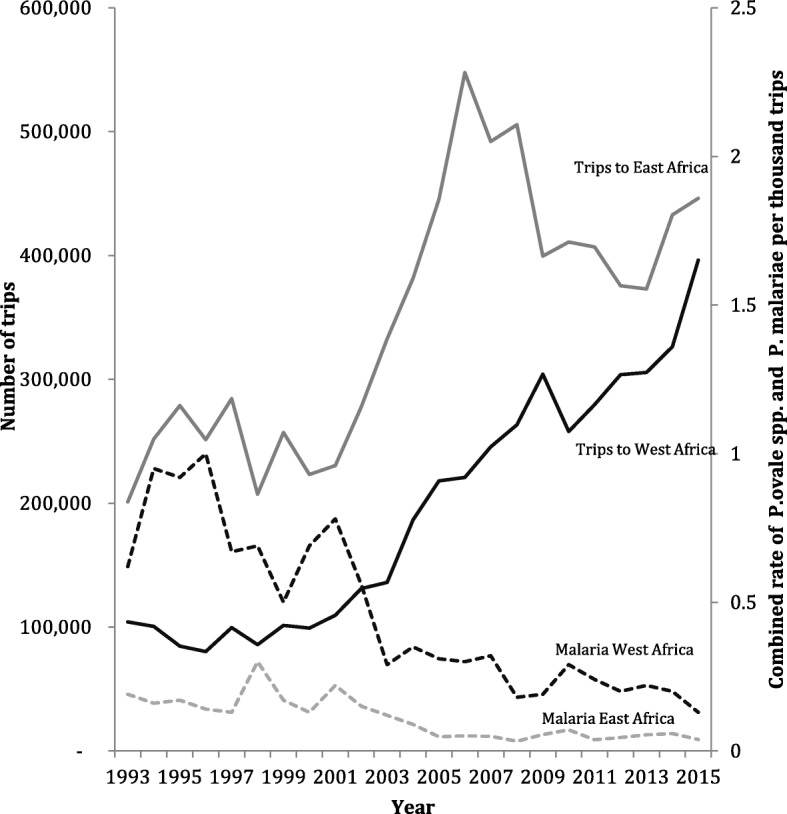
Incidence of *P. ovale* spp. and *P. malariae* per travel episode 1993–2015

Between 1993 and 2015, traveller numbers to West Africa increased by 380.6% whilst traveller numbers to East Africa increased by 221.9%. In West Africa, total attack rates of *P. ovale* spp. and *P. malariae* fell 4.8-fold from 0.62 to 0.13 per thousand trips. In East Africa, total attack rates fell from 0.19 to 0.038 per 1000 travel episodes, a fivefold reduction (Fig. 2).

### Latency of *P. ovale* spp. cases by malaria season

**Fig. 3:**
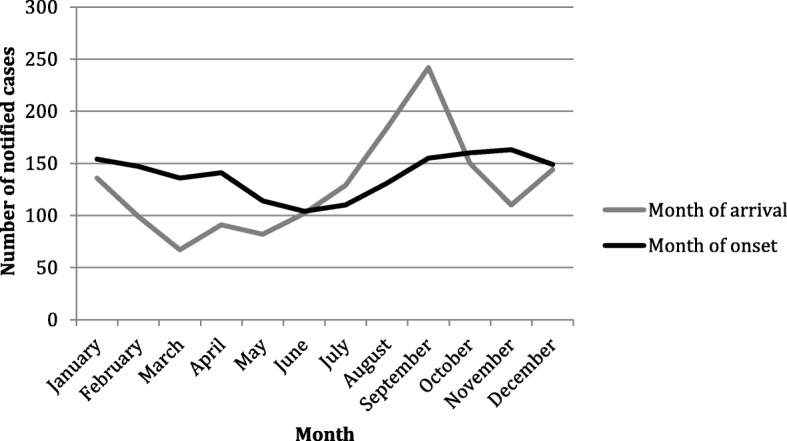
*P. ovale* spp. infection categorised by month of the UK arrival and month of onset of symptoms

Most patients with *P. ovale* spp. infection arrive in the UK between August and October but develop symptoms between September and January (Fig. 3). The overall median latent period was 76 days (IQ range 19–158 days, range − 53 to 3617 days), similar to that of *P. vivax* (67 days, (IQ range 9–211 days, range − 30 to 2195 days), which also has a hypnozoite state.

**Fig. 4:**
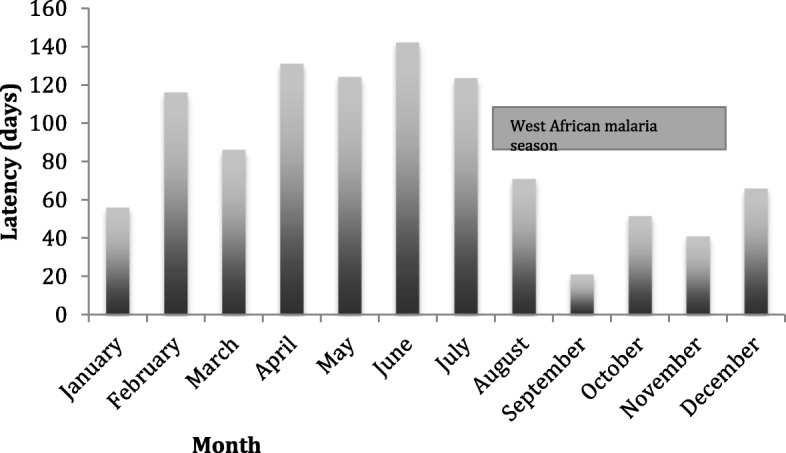
*P. ovale* spp*.* latency between arrival in the UK and onset of symptoms by month of symptom onset with relation to malaria season in West Africa

In patients who acquired infection in West Africa, there was a significant difference in latency between patients who presented with *P. ovale* spp. infection during the West African malaria season (44 days) compared to those presenting outside this season (94 days, *p* < 0.0001). There was no evidence of variation in latency by month of arrival. In patients who had acquired malaria in East Africa, there was no difference in latency between those presenting with symptoms during or outside the malaria season (73 days vs 87 days, *p* = 0.58) (Fig. 4).

For 243 patients, *P. ovale* spp. speciation data was available. 53.9% (131) patients had *P. ovale wallikeri* whilst 46.1% (112) had *P. ovale curtisi*. There were no cases shown to harbour both *P. ovale* spp. in our sample set. 65.6%(158) samples were from West Africa; 51.3% (81) were *P. ovale wallikeri* and 48.7% (77) were *P. ovale curtisi*. 22.4%(54) were from East Africa; 61.1% (33) were *P. ovale wallikeri* and 38.9% (21) were *P. ovale curtisi*. 11.5% (28) samples were from people who had travelled elsewhere in Africa. One sample (0.4%) was from a traveller to Southeast Asia, and 2 (0.85%) had unknown travel histories. *P. ovale curtisi* had a latent period of 86 days (IQ range 15–187 days, range − 3 to 538 days), significantly longer than that of *P. ovale wallikeri* at 29 days (IQ range 5–92 days, range − 8 to 558 days, *p* = 0.0014). The latent period in patients presenting with *P. ovale wallikeri* during the West African malaria season was significantly shorter at 11 days compared to those presenting outside this season (54 days, *p* = 0.0011). A similar trend was seen in *P. ovale curtisi*, but it did not achieve significance, in the context of small numbers (88 vs 41 days; *p* = 0.40) (Table 1).

**Table 1:** Latency in *P. ovale* spp. acquired in West Africa, malarial season vs non-malarial season

	Latency (days)	Latency in malaria season (days)	Latency in non-malaria season (days)	Significance (*p*)
*P. ovale* (*n* = 1599)	76	44	94	< 0.0001
*P. ovale curtisi* (*n* = 77)	80.5	41	88	0.4
*P. ovale wallikeri* (*n* = 81)	17.5	11	54	0.0011
Significance (*p*)	0.0073			

## Discussion

To our knowledge, this is the largest study in the literature to date of imported *P. ovale* spp. and *P. malariae* infection. It extends previous findings from the UK MRL [5, 6, 20]; the scale and duration of this study allows for robust clinical and public health findings. A striking finding is that *P. ovale* spp. infection tends to present during the West African high transmission malaria season, suggesting latency correlates with the peak transmission season in region of acquisition. Both species are uncommon causes of malaria in the UK, and almost all cases are imported from Africa. Incidence per travel episode is falling, probably due to improved malaria control in endemic areas, but on the background of steadily rising travel to malaria-endemic Africa, they are likely to remain an important if relatively rare cause of imported febrile illness for the foreseeable future. Although delayed presentation does occur in both *P. ovale* spp. and *P. malariae* infection, these data suggest that substantially delayed presentation is relatively rare. The very low mortality for both species in the context of a high-income setting is confirmed.

The most striking finding is the seasonal nature of *P. ovale* spp. latency between leaving West Africa and onset of symptoms. The latent period is significantly shorter in those presenting from West Africa with symptoms in Europe during the West African malaria season than outside it. Most delayed cases of *P. ovale* spp. malaria are likely to represent hypnozoite relapse, and this suggests that in West Africa, where malaria transmission is usually highly seasonal, relapse synchronises with the malaria season wherever it is acquired. This is of potential evolutionary benefit as it maximises the chance of parasite transmission to mosquito and subsequent human host. Relapsing forms of malaria are especially well adapted to getting through periods of lower transmission.

There are also public health implications of this finding; the peak in *P. ovale* spp. infection during the West African malaria season is likely to be larger and made up of increased transmission, but also increased relapse. Targeting transmission control measures during the malaria season may have a smaller effect on cases than would be expected if relapse happened randomly through the year. Conversely, targeting anti-hypnozoite interventions just before the West African malaria season may have a larger effect than would be predicted if randomness of relapse is assumed. The use of seasonal malaria chemoprevention, which targets malaria blood stages, may have a greater impact on *P. ovale* malaria because of this temporality.

A similar but stronger trend in latency has previously been reported in *P. vivax* which also has a hypnozoite stage. In the UK, *P. vivax* is predominantly acquired in the Indian sub-continent. Most patients present with *P. vivax* during the summer months in the UK, which coincides with the malaria season in the Indian sub-continent [21, 22]. The latent period is significantly shorter in people arriving in the UK during this period [20]. There has been speculation that *P. vivax* reactivation is due to increased ambient temperatures in the UK. However, the comparable trend that we have observed with *P. ovale* spp. lends weight to the hypothesis that relapse occurs in the malarial season. Thus, there is evidence that each malaria species with hypnozoite stages relapses during the peak malaria transmission season in their region of acquisition, if transmission in that area is highly seasonal.

The clear season-dependent difference in latency for *P. ovale* spp. infection imported from West Africa was not observed in cases imported from East Africa. The seasonality of malaria transmission is generally much less pronounced in East Africa, one reason seasonal malaria chemoprevention is less appropriate in that area [13]. As 98% of *P. ovale* spp. imported to the UK comes from Africa, it was not possible to interpret this trend for other regions of the world.

*P. ovale curtisi* and *P. ovale wallikeri* have previously been found to be sympatric in West and East Africa and are proposed to have significantly different latent periods [6, 9]. This is confirmed in the current larger study where median latencies were estimated at 86 and 23 days respectively. For *P. ovale wallikeri*, the latent period was significantly shorter in those presenting with symptoms during the West African malaria season than outside it (11 vs 54 days). A similar trend was visible in *P. ovale curtisi* but did not reach significance, possibly due to small numbers; the two species were not differentiated until recently so the numbers of cases available for analysis were few. Alternatively, it may be that the overall correlation seen with *P. ovale* spp. is driven solely by *P. ovale wallikeri* and that this seasonal relationship does not exist for *P. ovale curtisi*. Although the hypnozoite state has not been formally identified in either of these species, these data suggest that it exists and that hypnozoites are programmed to activate in the season when they are most likely to be transmitted to a mosquito vector.

Interestingly, the relative abundance of *P. ovale* spp. among travellers compared to *P. malariae* is the reverse of reports from cross-sectional studies in the field, where infections with *P. malariae* are more likely to be observed. This interesting discrepancy may be due to a higher mosquito transmission capacity for ovale malaria, and a longer duration of infection for *P. malariae* [10].

As in other studies, mortality from both *P. ovale* spp. and *P. malariae* infection is low [23, 24]. These large numbers give confidence in supporting the UK and other international guidance that these infections can usually be managed in outpatient settings unless the patient has significant comorbidities or is unable to tolerate oral therapy [25]. In contrast to *P. falciparum* and *P. vivax*, older age was not associated with increased mortality in these imported infections [20, 26]. However, the long delay in some cases between leaving an endemic area and development of illness presents a diagnostic challenge. It is essential that clinicians take an extended travel history and consider malaria in 2 years after people return from Africa.

The strength of this study lies in its large size and use of national surveillance data, collected prospectively with little methodological change, over a period of 29 years. As these species are relatively much less frequent causes of imported malaria, only large datasets can explore them. Because the UK is a non-endemic area, we are able to draw conclusions on latent periods and seasonal trends as the possibility of reinfection can be excluded. These trends would not be possible to establish in endemic zones due to continuous exposure and the possibility of reinfection.

There are a number of limitations, common to all studies using national observational data. The database relies on passive case detection and, inevitably, will underestimate the numbers of imported cases although capture-recapture data suggest the majority are identified. It does not capture data on the UK residents taken ill and treated for malaria whilst travelling abroad. We defined the latent period as time from arrival in the UK to onset of symptoms. This definition underestimates the true latent period as it is unlikely travellers are infected on the day that they left an endemic area. However, this caveat is very unlikely to explain the temporal trend of *P. ovale* spp. cases. Unfortunately, duration of individual travel episodes was not collected in a sufficiently uniform way to be able to use it reliably in analysis.

Although it has been a routine to distinguish *P. ovale curtisi* from *P. ovale wallikeri* in samples of *P. ovale* received in the MRL since 2013, limited speciation data was available from samples before this period. There is increasing evidence, from this and other studies, that these two species behave differently regarding latency. We were only able to analyse this in a small subset of samples.

## Conclusion

*P. ovale* spp. and *P. malariae* are unusual causes of imported malaria to the UK and are almost always acquired in Africa. Incidence per travel episode is falling, and mortality rates are very low. Whilst *P. malariae* usually presents clinically within the first 28 days after return, the latent period of *P. ovale* spp. is significantly longer at 67 days. In travellers returning from West Africa, there is a significant difference in latency in patients presenting with symptoms during the malaria season in the region from which they travelled compared to those presenting outside this season. This is similar to the trend seen in imported *P. vivax* and suggests that, in areas where malaria transmission is highly seasonal, hypnozoites from both species activate during the season when they are most likely to be transmitted to a mosquito vector and subsequent human host.

## Abbreviations

IPS: International Passenger Survey
IQ range: Interquartile range
MRL: Malaria Reference Laboratory
PCR: Polymerase chain reaction
PHE: Public Health England
UK: United Kingdom
